# The placebo effect and its determinants in fibromyalgia: meta-analysis of randomised controlled trials

**DOI:** 10.1007/s10067-017-3595-8

**Published:** 2017-03-15

**Authors:** Xi Chen, Kun Zou, Natasya Abdullah, Nicola Whiteside, Aliya Sarmanova, Michael Doherty, Weiya Zhang

**Affiliations:** Arthritis Research UK Pain Centre, Academic Rheumatology, Division of Rheumatology, Orthopaedics and Dermatology, University of Nottingham, Nottingham, UK

**Keywords:** Fibromyalgia, Meta-analysis, Pain management, Placebo effect, Rheumatic diseases, Systematic reviews and methodology

## Abstract

The aims of this study were to determine whether placebo treatment in randomised controlled trials (RCTs) is effective for fibromyalgia and to identify possible determinants of the magnitude of any such placebo effect. A systematic literature search was undertaken for RCTs in people with fibromyalgia that included a placebo and/or a no-treatment (observation only or waiting list) control group. Placebo effect size (ES) for pain and other outcomes was measured as the improvement of each outcome from baseline divided by the standard deviation of the change from baseline. This effect was compared with changes in the no-treatment control groups. Meta-analysis was undertaken to combine data from different studies. Subgroup analysis was conducted to identify possible determinants of the placebo ES. A total of 3912 studies were identified from the literature search. After scrutiny, 229 trials met the inclusion criteria. Participants who received placebo in the RCTs experienced significantly better improvements in pain, fatigue, sleep quality, physical function, and other main outcomes than those receiving no treatment. The ES of placebo for pain relief was clinically moderate (0.53, 95%CI 0.48 to 0.57). The ES increased with increasing strength of the active treatment, increasing participant age and higher baseline pain severity, but decreased in RCTS with more women and with longer duration of fibromyalgia. In addition, placebo treatment in RCTs is effective in fibromyalgia. A number of factors (expected strength of treatment, age, gender, disease duration) appear to influence the magnitude of the placebo effect in this condition.

## Introduction

Fibromyalgia (FM) is a common chronic multiple regional pain syndrome, affecting approximately 2% of people in the general population [1]. The condition is eight times more common in women than in men [2] and increases with age [3]. The main symptoms of fibromyalgia include widespread pain, fatigue, non-restorative sleep, and cognitive impairment [4]. Treatments for fibromyalgia include non-pharmacologic therapies such as patient education, aerobic exercise, acupuncture and cognitive behavioural therapy, and pharmacologic therapies such as analgesics and anti-depressants. Placebo has been proved to be effective in many conditions such as depression [5] chronic fatigue syndrome [6], Parkinson’s disease [7], irritable bowel syndrome [8] and osteoarthritis (OA) [9]. The magnitude of placebo effect is known to be influenced by a number of factors [10, 11]. In OA, placebo appears to be more effective in subjective outcomes such as self-reported pain, function, stiffness and physician global assessment [12]. A single systematic review in 2011 attempted to identify the magnitude and patient-specific predictors of placebo response in FM, and the year of study initiation, pain baseline, and effect size in active drug groups were found to be associated with the placebo response [13]. However, whether a placebo is effective in FM and what the determinants are of any such effect remain unknown.

## Methods

### Literature search

A systematic literature search was carried out up until October 2015, using Medline (1950–present), Web of Science (1960–present), EMBASE (1980–present), Cumulative Index to Nursing and Allied Health Literature (CINAHL) (1982–present), and Allied and Complementary Medicine (1985–present). The search strategies included: [1] search for FM; [2] search for randomised controlled trials (RCTs). Other terms that were used for FM and RCTs such as chronic widespread pain, fibrositis, double blind method, single blind method and comparative study were also included (Appendix 1). There were no language restrictions for this search. Citations were imported into Endnote X7 and duplications were removed. Titles and abstracts were read to judge whether the studies met the inclusion criteria. Full papers were obtained for further scrutiny of relevant studies according to the inclusion/exclusion criteria.

### Inclusion and exclusion criteria

Only RCTs with placebo controlled groups and/or no-treatment control groups in FM or chronic widespread pain were included. Trials that only compared two active treatments or which studied several chronic pain conditions without clear definition or separation of FM/chronic widespread pain were excluded. Trials that did not have any clinical outcomes were also excluded.

### Data extraction

A data extraction form was developed to collect data from each of the included studies. The following information was extracted from each study: study design (parallel or cross-over), study setting (hospital or community), funding body (industry, charity or academic), sample size (treatment group and control group), mean age of participants and standard deviation (SD), percentage of women participants, and outcome measures at both baseline and endpoint.

### Outcome measures

Primary outcomes of the study included pain, fatigue, sleep quality and physical function. The outcomes were decided before the literature search in order to get the relevant studies. After reviewing all retrieved studies, the visual analogue scale (VAS) was found commonly used to measure pain reduction. In fatigue measurement, the Multidimensional Fatigue Inventory (MFI) was the most commonly used tool. MFI is a 20-item self-report instrument designed to measure fatigue that covers the following dimensions: general fatigue, physical fatigue, mental fatigue, reduced motivation and reduced activity [14]. Pain, fatigue and sleep quality measured by VAS were taken if reported. Other scales such as the Likert scale and categorical scale were used when VAS was not available. Standardised mean difference was calculated in the meta-analysis to avoid heterogeneity that was caused by the usage of different measurement tools for the same outcome.

Secondary outcomes included quality of life (QOL), participant and observer assessment of overall wellbeing, and others. The Fibromyalgia Impact Questionnaire (FIQ) and SF36 were normally used as disease-specific and generic QOL instruments respectively in FM. Other QOL measurements were also included.

### Quality assessment and data validation

Details of randomisation (yes/no/unknown), allocation concealment (adequate/inadequate/unknown), blinding to participants, care providers and assessors (yes/no/unknown), dropout (%), intention-to-treat analysis (yes/no/unknown), recruitment setting (community/hospital/unknown) and funding body (industry/non-industry/unknown) were assessed for each trial.

The data were first extracted independently by the main reviewer (X.C). Subsequently, a second reviewer (K.Z.) randomly selected 10% of the studies and extracted the data independently. Agreement was examined between the two data extractions. If a disagreement of more than 5% was found, the full data extraction was undertaken by the second reviewer. Disagreement was discussed and consensus was achieved with other reviewers (M.D. and W.Z.).

### Statistical analysis

Effect size (ES), that is the standardised mean difference (SMD), was calculated for each outcome measure. The ES standardises the difference using the pooled within group SD between baseline and endpoint. It therefore normalises the measures across studies which permits the combined analysis. However, unlike the natural measure of the outcome such as pain on a visual analogue scale (VAS–where pain severity is scored from 0 to 100 mm), ES measures the size of effect in the unit of SD. According to Cohen’s definition, an ES of 0.2 (i.e. 20% of SD) suggests a small effect, an ES of 0.5 (i.e. 50% of SD) indicates a moderate effect, and an ES of 0.8 (80% of SD) or more is a large effect [15]. Hedges (1982) method was used to calculate ES and its 95% confidence intervals (CI) to further adjust for sample size [16]. ES from baseline to the endpoint was calculated for each study arm. Comparison was made between placebo and no-treatment groups to establish the placebo effect on the basis of no overlapping between the two 95%CIs. A funnel plot (Appendix 2) was used to examine the possibility of publication bias in each analysis. The Egger’s test was used for asymmetry of the funnel plot [17]. *I* ^2^ (a measure for inconsistency among studies ranging from 0 to 100%) was used to measure heterogeneity [18]. The larger the *I* ^2^, the greater the inconsistency or heterogeneity of study result. The Q test was applied to determine whether any heterogeneity was statistically significant [19]. Subgroup analysis was undertaken according to treatment and trial characteristics.

## Results

### Characteristics of the trials

**Fig. 1:**
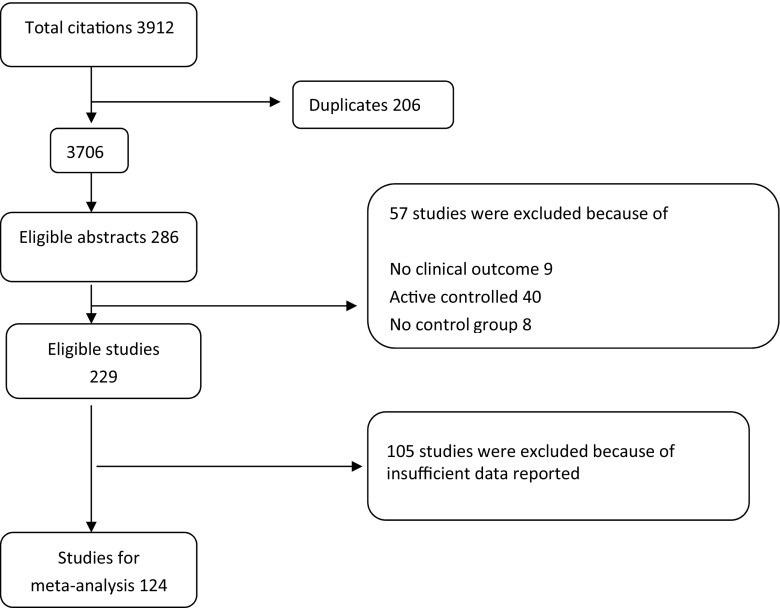
Flowchart of literature search

In total, 3912 citations were retrieved from all databases. After removing duplicates, 3706 citations remained. After reading abstracts, 286 studies were found relevant. Full studies were retrieved and examined. Subsequently, 229 of these met the inclusion criteria. In order to perform meta-analysis, both the means and SDs of each outcome measure are needed. Trials that failed to provide suitable data were not included in the quantitative meta-analysis. A total of 105 eligible trials could not be included in the analysis because of the lack of suitable data. Finally, 124 trials remained for meta-analysis (Fig. 1).

A total of 15,633 participants were included in the 124 trials. More trials used placebo as a comparator than a no-treatment control (73 vs 51). The demographic characteristics (mean age, percentage of women, etc.) of the participants in placebo groups did not different from that of the no-treatment groups. The majority of the trials (70%) reported pain as an outcome. Other commonly reported outcomes were fatigue, sleep quality and physical function (Table 1).

**Table 1:** Demographic Characteristics of Included Trials

	Total	Control	
		Placebo	Untreated
No. of trials	124	73	51
No. of participants	15,633	12,041	3592
Mean age, range (year)	49.2 (29.4 to 59.0)	49.0 (29.4 to 59.0)	49.4 (40.8 to 58.5)
Women (0–100) %	95.4 (63.7 to 100)	94 (63.7 to 100)	100 (74 to 100)
Percentage of trials reporting			
Pain		70%	72%
Fatigue		41%	32%
Sleep disturbance		25%	26%
Physical function		19%	30%
FIQ total score		45%	62%
BDI total score		12%	17%
No. of tender sites		32%	30%

*FIQ* Fibromyalgia Impact Questionnaire, *BDI* Beck’s Depression Index

The funnel plot of ESs for pain reduction from baseline in either placebo or no-treatment groups showed an asymmetric distribution (Egger’s *p* = 0.04), i.e. trials with smaller ES with placebo or no-treatment control groups were more likely to be published.

### Efficacy of placebo versus no treatment

**Fig. 2:**
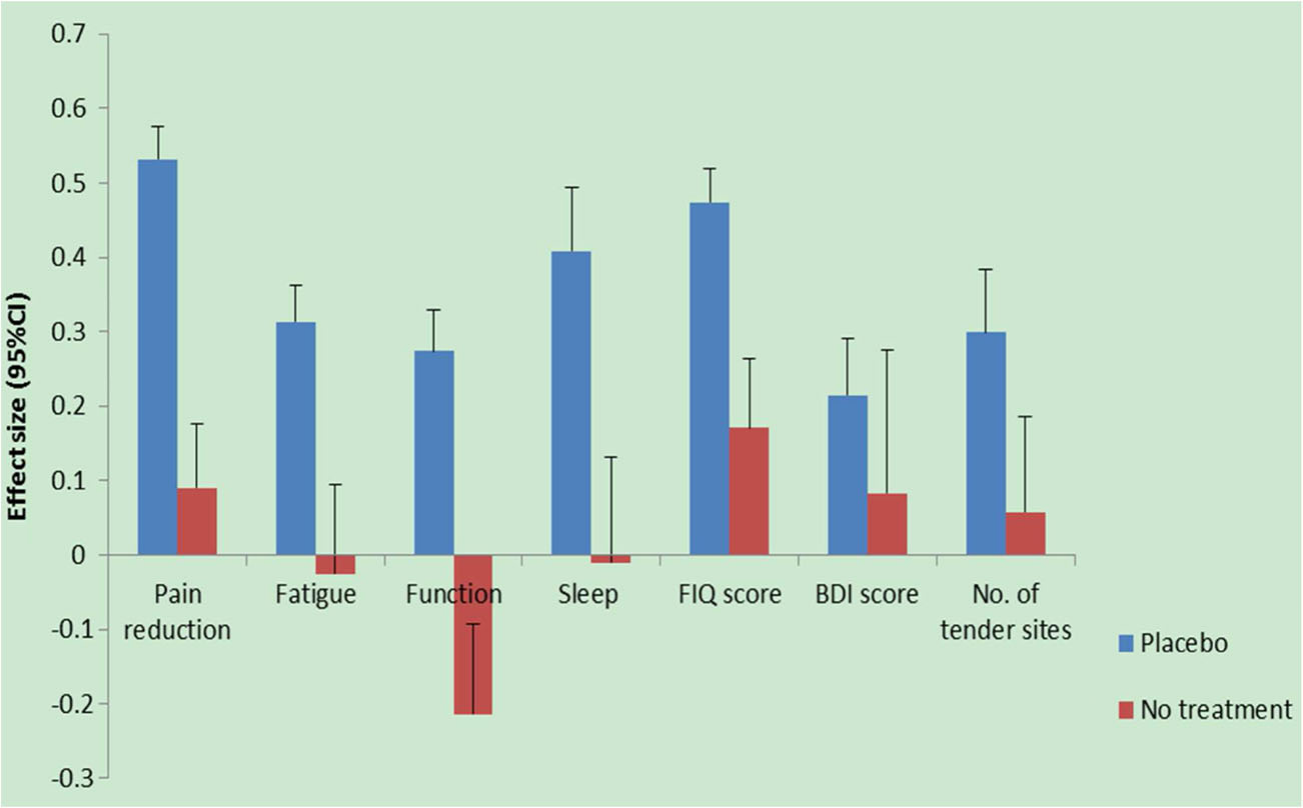
Comparison of placebo group and the untreated group *FIQ* Fibromyalgia Impact Questionnaire; *BDI* Beck’s Depression Index

Compared with the ESs of no-treatment (observation only) groups, placebo was statistically superior to no treatment in all outcomes of FM, apart from Beck’s Depression Index (BDI) and number of tender sites (Fig. 2). The magnitude of placebo effect was clinically moderate in pain reduction, (ES = 0.52, 95%CI 0.48 to 0.57). The ES varied from study to study. Heterogeneity in these studies was high (*I* ^2^ = 74.2%, *P* < 0.0001). Similar results were observed in other outcomes. The results were summarised in Table 2.

**Table 2:** Placebo Effect by Outcome Measures in Fibromyalgia

Outcome	No. of trials	No. of participants	Pooled effect size (95%CI)	Publication bias (Egger)	Heterogeneity (*I* ^2^)
Pain reduction	51	4472	0.52 (0.48, 0.57)	−0.95, *p* = 0.04	74%, *p* < 0.0001
Fatigue	30	3465	0.31 (0.26, 0.36)	−0.30, *p* = 0.45	25%, *P* = 0.1078
Physical function	14	2435	0.27 (0.22, 0.33)	−1.47, *p* = 0.05	46.7%, *p* = 0.0276
Sleep quality	18	1048	0.41 (0.32, 0.49)	−0.90, *p* = 0.07	0%, *p* = 0.5195
FIQ total score	33	3897	0.47 (0.43, 0.49)	0.19, *p* = 0.76	64.3%, *p* < 0.0001
BDI total score	9	1504	0.21 (0.14, 0.29)	0.06, *p* = 0.95	51.7%, *p* = 0.0351
No. of hyperalgesic tender sites	23	1120	0.30 (0.21, 0.38)	1.58, *p* = 0.24	84.4%, *p* < 0.001

### Determinants of the placebo effect

In the subgroup analysis, the placebo ES increased with the mean age of participants in a trial. The placebo ES was 0.42 (95%CI 0.16 to 0.68) for people under 40 years, 0.51 (95%CI 0.47 to 0.56) for people aged 40–50 years, and 0.68 (95%CI 0.57 to 0.79) for those aged over 50 years, respectively. With respect to gender, the more women included in a trial, the less effective was placebo. Trials with <80% women had a placebo ES of 0.65 (95%CI 0.32 to 0.98), whereas trials with 100% women had a placebo ES of 0.21 (95%CI 0.02 to 0.39).

Studies of participants with a shorter mean duration of FM (3–7 years) tended to have a larger placebo ES (0.59, 95%CI 0.51 to 0.76) than those with longer mean duration of FM (over 13 years) (0.26, 95%CI -0.09 to 0.60). Baseline pain severity also appeared to influence placebo ES. Groups with the lowest mean baseline pain severity (<60%) had the smallest placebo effect (ES 0.22, 95%CI 0.06 to 0.42) and there was a tendency for increasing placebo effect with higher baseline pain severity (Table 3).

**Table 3:** Subgroup analysis for possible determinants

	No. of trials	No. of patients	ES (95%CI)	Heterogeneity (*I* ^2^)	*P*_Publication bias_
Mean age (years)					
<=40	5	115	0.42 (0.16, 0.68)	66.1%, *p* = 0.02	0.60
>40, <=50	30	3474	0.50 (0.45, 0.55)	67.3%, *p* < 0.0001	0.81
>50	12	711	0.62 (0.53, 0.70)	82.7%, *p* < 0.0001	0.03
Women%					
<80%	4	77	0.65 (0.32, 0.98)	52.6%, *p* = 0.09	0.86
>80%, <90%	7	500	0.48 (0.35, 0.61)	64%, *p* = 0.01	0.50
>90%,<100%	22	3342	0.56 (0.52, 0.61)	82.4%, *p* < 0.0001	0.78
100%	9	277	0.21 (0.02, 0.39)	42.2%, *p* = 0.09	0.60
Mean disease duration					
3–7 years	13	1237	0.59 (0.51, 0.67)	76.3%, *p* < 0.0001	0.30
8–12 years	9	1208	0.57 (0.49, 0.65)	88%, *p* < 0.0001	0.46
13 years	2	65	0.26 (−0.09, 0.60)	N/A*	N/A*
Baseline pain severity (%)					
<60	8	195	0.22 (0.06, 0.42)	32.26, *p* = 0.1657	0.98
60–70	23	3025	0.54 (0.49, 0.59)	75.3, *p* < 0.0001	0.43
>70	15	1147	0.56 (0.48, 0.65)	79.9%, *p* < 0.0001	0.40

**Fig. 3:**
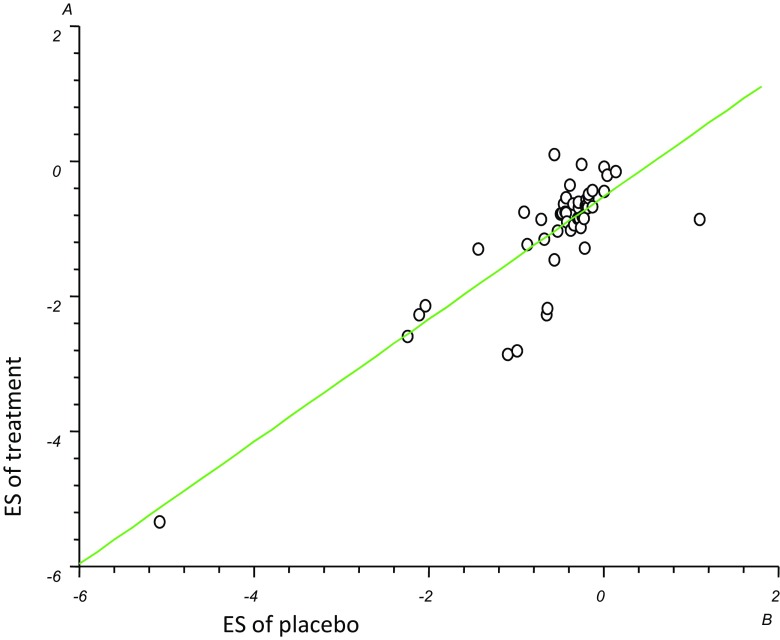
Correlation between effect size of active treatment and placebo *r* ^2^ = 0.703821, *P* < 0.0001

In addition, the placebo ES correlated significantly with the treatment ES (*r* = 0.70, *p* < 0.0001) (Fig. 3), i.e. placebo effect increased with the strength of active treatment.

Other potential determinants of the placebo effect were examined, including study setting (community, hospital), and blinding. However, due to the limited resources, no clear tendency was found.

## Discussion

The literature search identified over 200 placebo-controlled trials examining a wide range of different treatments for FM including drugs, physical interventions such as exercise and balneotherapy, psychological treatments such as CBT, and complementary therapies such as homeopathy and acupuncture. Some trials used placebo as the control while others used a no-treatment (observation only) group as a comparator. This provided an excellent opportunity to investigate the placebo effect and its determinants in FM. The study yielded three key findings. Firstly, participants receiving placebo treatment in RCTs obtain significant improvements in all the main outcome measures such as pain, fatigue, sleep quality, function and overall wellbeing. Secondly, these effects are superior to any changes observed in control groups who received no treatment. Thirdly, the magnitude of the placebo effect increases with the effect size of the active treatment, and is greater in people with more severe symptoms, in those with shorter duration of FM, in men more than women and in older compared to younger people with FM.

The debate concerning whether placebo treatment works for medical conditions has continued since Beecher’s landmark paper in 1955 entitled “The powerful placebo” [20]. Many studies suggest that the placebo response in RCTs highlighted by Beecher may be explained by spontaneous changes such as the natural variation of disease and regression to the mean [21–24]. Therefore, the comparison between placebo and no-treatment observational groups is essential to confirm whether placebo is effective or not [25]. We undertook such a comparison and found that receiving a placebo is significantly better than receiving no treatment for pain and other patient-centred outcomes of FM such as sleep, fatigue, function and overall quality of life. This finding is in line with a systematic review of RCTs of treatments in OA which found placebo treatment to be clinically effective for pain (ES, 0.51, 95%CI 0.46 to 0.55) and other outcomes of OA [10].

The observation that placebo ES goes up with the strength of active treatment is consistent with the recognised importance of patient expectancy for treatment in determining the magnitude of placebo effect [26, 27]. A positive association between severity of baseline pain and magnitude of placebo effect, as found in our study, has also been reported in OA RCTs [12]. This also accords with the observation in Parkinson’s disease that patients with greater baseline functional impairment are more likely to have a higher placebo effect [28]. The lower placebo ES observed in trials of participants with longer mean duration of FM accords with the observation that early intervention in FM is more likely to give a good outcome [28]. This implies that the longer a person has FM, the more entrenched the condition becomes, the lower the patient expectancy and the harder it is to improve outcomes by either active treatment or placebo and by other factors that influence contextual response.

Gender has been suggested previously to influence placebo response [29] with men appearing to be better placebo responders in some studies [30, 31]. The finding of the present study is in agreement with this. Pain physiology differs between men and women, and it is possible that ability to influence descending inhibition of pain through expectancy differs constitutionally between men and women [32, 33]. This is particularly pertinent in that women are more likely to have FM and other chronic pain than men, and the management of these types of pain is more challenging in women [34, 35]. One possible explanation is that women perceive disease differently from men [36]. Women may perceive an excess of symptoms compared with men, because of greater attention and increased attribution of bodily sensations to physical illness [37]. Another reason is that men may be better responders to treatments in general [38]. For example, in patients with Achilles tendinopathy, eccentric training results in significantly greater pain reduction in men than women [38]. Men also respond better than women to non-painful conditions, such as pulmonary arterial hypertension [39] and growth hormone deficiency [40]. The role of personality trait might also cause a gender difference in placebo response. High dispositional optimism and low state anxiety have been found to be significant predictors of placebo response and they associate with male gender [41].

A greater placebo response in older people has been reported in previous studies. According to Alexopoulos, more than half of elderly people with depression show at least 25% improvement from taking placebo [42]. In trials in Parkinson’s disease older individuals also show a significantly greater response to placebo [28]. Older people with FM also responded better to placebo in the current review. This could be explained by the fact that people of different ages have difference illness perceptions and expectancy. Although the prevalence of FM goes up with age, older people report less severe symptoms [43]. They may also be more likely to regard pain as part of the ageing process, to have less anxiety associated with this, and to cope with it better [43]. Whether this age-related placebo effect in FM is related to experience, social ability and other contextual factors deserves further study.

There are a number of caveats to this study. Firstly, the included trials may not cover all eligible trials in FM, especially those that are unpublished. Furthermore, many included trials did not present their result in numerical data and could not be used for the meta-analysis. Secondly, inclusion of many disparate treatments and their placebo in this review resulted in high heterogeneity for the placebo effect. Although we carefully considered the reasons for marked heterogeneity and undertook a number of subgroup analyses, the heterogeneity still remained for some subgroups and a random effects model had to be used to pool the data. Thirdly, the determinants were examined by individual factors in subgroup analysis. Therefore, only a brief tendency can be determined. Further study of the determinants for the placebo effect is warranted. Furthermore, the influence of gender and age on the placebo effect would best be studied using individual participant data rather than a meta-analysis of trials where only the gender proportion and mean ages are available in each trial.

In conclusion, placebo treatment appears to be clinically effective in FM in reducing pain and fatigue and improving non-restorative sleep and overall quality of life. Its analgesic effect is clinically significant. Greater placebo effect may be observed in men, in older people and in those with more severe pain, whereas a lower placebo effect is seen in those with longer established FM. Further studies using individual participant data are required to better identify the individual predictors of placebo effect size.

## Appendix 1. Searching strategy

### Medline OVID 1948–present

1. exp Meta-Analysis/
2. systematic review.mp.
3. quantitative review.mp.
4. quantitative overview.mp.
5. statistical pool.mp.
6. 1 or 2 or 3 or 4 or 5
7. exp Randomized Controlled Trial/
8. exp Clinical Trial/
9. exp Double-Blind Method/
10. exp Single-Blind Method/
11. Comparative Study/
12. 7 or 8 or 9 or 10 or 11
13. exp Fibromyalgia/
14. fibromyalgia syndrome.mp.
15. Chronic widespread pain.mp.
16. Fibrositis.mp. or Fibromyalgia/
17. 13 or 14 or 15 or 16
18. 12 and 17
19. 6 and 17
20. 18 or 19
